# Identification of *Helicobacter* *pylori*-related gastric cancer risk using serological gastritis markers and endoscopic findings: a large-scale retrospective cohort study

**DOI:** 10.1186/s12876-022-02381-z

**Published:** 2022-06-20

**Authors:** Naoko Nagasaki, Masanori Ito, Tomoyuki Boda, Takahiro Kotachi, Hidehiko Takigawa, Shiro Oka, Shinji Tanaka

**Affiliations:** 1 Department of Gastroenterology and Metabolism, Hiroshima University Hospital, Hiroshima, Japan; 2 Department of General Internal Medicine, Hiroshima University Hospital, Hiroshima, Japan; 3 Department of Internal Medicine, Hiroshima Memorial Hospital, Hiroshima, Japan; 4 Department of Endoscopy, Hiroshima University Hospital, 1-2-3 Kasumi, Minami-ku, Hiroshima, Hiroshima, 734-8553 Japan

**Keywords:** ABC method, Gastric cancer, Endoscopy, Gastritis markers, *Helicobacter pylori*

## Abstract

**Background:**

Gastric cancer remains a severe public health problem worldwide, particularly in Japan. Recent studies have demonstrated that serum markers are beneficial for risk stratification in gastric cancer development. We aimed to evaluate the usefulness of serum markers either alone or in combination (serum markers plus endoscopy) for effective risk stratification of gastric cancer development.

**Methods:**

We enrolled 22,736 patients aged 20–95 years who underwent blood sampling and endoscopic examination at Hiroshima University Hospital in Japan between 1990 and 2014. The serum pepsinogen (PG) levels and anti-*Helicobacter pylori* antibody (Hp-Ab) titers were evaluated in each patient. The enrolled patients were matched with the database of the Hiroshima Prefecture Regional Cancer Registry. We processed the medical records and excluded patients with possible confounding factors for PG levels, such as proton pump inhibitor use, prior successful eradication therapy, post-gastrectomy, severe hepatorenal dysfunction, Zollinger–Ellison syndrome, and autoimmune gastritis. Among the remaining 5131 patients, we reviewed records of endoscopic examinations and selected 1507 patients (mean age, 62.5 years; 985 men and 522 women) who underwent endoscopic examination more than three months after blood sampling.

First, based on the ABC method, patients were classified as follows: High PG levels and negative Hp-Ab, group A, high PG levels and positive Hp-Ab, group B, low PG levels and positive Hp-Ab, group C, and low PG levels and negative Hp-Ab, group D. Group A was further classified into two subgroups using endoscopic findings: true A without atrophic gastritis and pseudo A with atrophic gastritis. All patients underwent annual endoscopy follow-up.

**Results:**

Among the 1,507 patients (mean age, 62.5 years; 985 men), 24 were diagnosed with newly developed gastric cancer. No significant difference in cancer development was found between group A (PG negative and Hp-Ab negative) and the other groups. Remarkably, no true A group subjects developed gastric cancer.

**Conclusions:**

The combination of serum markers and endoscopic findings is essential for the risk evaluation of gastric cancer.

**Supplementary Information:**

The online version contains supplementary material available at 10.1186/s12876-022-02381-z.

## Background

Gastric cancer is the fifth most common cancer and the fourth most common cause of cancer death worldwide [1]. Most cases of non-cardia gastric cancer are caused by *Helicobacter pylori* infection [2, 3]. Uemura et al. reported that gastric cancer develops in individuals infected with *H. pylori* but not in uninfected people [4]. The incidence of gastric cancer in Japan has been declining because of improvements in the public health system and increases in insurance coverage for eradicating *H. pylori*-induced gastritis. However, it is still high relative to the global incidence [3].

Mass screening using endoscopic examination has reduced gastric cancer mortality rates [5–8]. Endoscopy is currently recommended as a part of the healthcare public policy in Japan. Nevertheless, it is important to perform risk stratification of gastric cancer for effective screening of healthy individuals since endoscopic examination is associated with a risk of adverse events [9].

Serum pepsinogens (PGs) are representative biomarkers of mucosal conditions, such as inflammation and atrophy in the stomach, and the PG method using PG I and PG I/II is useful for risk stratification of gastric cancer [10–16]. In addition, the ABC method, which is performed by evaluating serum anti-*H. pylori* antibody (Hp-Ab) titers and serum PG levels are also useful methods for risk stratification of gastric cancer [17–20]. In this system, group A patients, defined as those with negative results in the Hp-Ab or PG method, are regarded as *H. pylori*-uninfected and have little risk for gastric cancer [18, 19]. However, in clinical practice, gastric cancer is sometimes diagnosed in patients classified into group A [21]. The main reason for this discrepancy may be that group A includes some previously infected patients with *H. pylori* [22]. The Japanese Society of Helicobacter Research recently published an advanced flowchart for the risk stratification of gastric cancer, which comprises serum tests and endoscopic examinations; a low Hp-Ab titer without endoscopic atrophy on screening endoscopy indicates little or no risk of gastric cancer [23].

This study aimed to evaluate serum markers alone (ABC method based on PG levels and Hp-Ab titers) or in combination (ABC method plus endoscopy) for effective risk stratification of gastric cancer development in Japanese patients using a large-scale clinical database.

## Methods

### Patients

We enrolled 22,736 patients who underwent endoscopic examination and serum marker evaluations at Hiroshima University Hospital, Hiroshima, Japan, between 1990 and 2014. First, we removed duplicate data and excluded patients with missing values. We checked the medical records, and 8448 patients were matched with the Hiroshima Prefecture Regional Cancer Registry Database by name, sex, address, and birth date. In this database, the death certificate notification, death certificate only, and incidence mortality ratio were 6.7%, 4.1%, and 2.53, respectively.

We processed the medical records and excluded patients with possible confounding factors for PG levels, such as proton pump inhibitor use, prior successful eradication therapy, post-gastrectomy, severe hepatorenal dysfunction, Zollinger–Ellison syndrome, and autoimmune gastritis. Among the remaining 5131 patients, we reviewed records of endoscopic examinations and selected 1507 patients (mean age, 62.5 years; 985 men and 522 women) who underwent endoscopic examination more than three months after blood sampling. For cancer-free patients, we considered the latest day of endoscopic examination without findings of gastric cancer as a censored case.

### Evaluation of serum markers

Fasting blood samples were collected from participants, and the serum samples were stored at − 20 °C until use. Serum PG I and PG II levels were measured by radioimmunoassay (Abbott, Tokyo, Japan) from 1990 to 1999, chemiluminescent immunoassay (Abbott) from 1999 to 2001, enzyme immunoassay (E-plate test; Eiken, Tokyo, Japan) from 2001 to 2003, and latex agglutination test (L-Z test; Eiken) from 2003 to 2014. Patients with PG I levels > 70 ng/mL or PG I/PG II levels > 3 ng/mL were considered to have high PG levels [13]. Serum Hp-Ab titers were evaluated using an enzyme-linked immunosorbent assay (E-plate; Eiken). The cut-off value was set to 3 U/mL [24]. Using the definition of the ABC method, we classified patients with high PG levels and negative Hp-Ab, high PG levels and positive Hp-Ab, low PG levels and positive Hp-Ab and low PG levels and negative Hp-Ab, into groups A, B, C and D, respectively [13].

### Endoscopic diagnosis of atrophic gastritis

The status of atrophic gastritis in each patient was assessed by endoscopic evaluation based on the Kimura–Takemoto classification [25]. The absence of endoscopic atrophic changes in the gastric corpus was defined as atrophic gastritis (C-0 or C-1 in the Kimura–Takemoto classification). Group A patients were further classified into true A (without atrophic gastritis) and pseudo A (with atrophic gastritis) subgroups. The definition of true A is negative results for both endoscopic atrophy and serological markers.

### Statistical analysis

The *χ*^2^-test and Fisher’s exact test were used to compare categorical data. The Cox proportional hazards regression model or log-rank test with Kaplan–Meier analysis was used to assess the differences in cancer development among the subtypes. Confidence intervals were computed using the normal approximation of the binomial distribution. A *P*-value < 0.05 was considered indicative of statistical significance for each test. JMP statistical software (SAS Institute Inc., Cary, NC, USA) was used for all calculations.

## Results

### Final patients enrolled in this study

We enrolled 22,736 patients, of which 8448 were matched with the regional tumor registry data in the Hiroshima Prefecture. Finally, 1507 patients who underwent endoscopic examinations more than three months after blood sampling were included. Among the 1507 patients, 24 were diagnosed with newly developed gastric cancer. The clinicopathological data of the 24 patients with gastric cancer are summarized in Table 1.

**Table 1:** Clinicopathological findings of all 24 cases of gastric cancer

Characteristic	Cases of cancer (n = 24)
*Sex, n (%)*	
Men/Women	22 (92)/2 (8)
Age, years, median (range)	63.5 (45–81)
*Group*	
Pseudo A/B/C/D	2/7/12/3
*Gastric location, n (%)*	
Upper	2 (8)
Middle	12 (50)
Lower	10 (42)
*Histological type, n*	
Adenocarcinoma	24
Tubular adenocarcinoma	18
Papillary adenocarcinoma	2
others	4

### Relationship between the ABC method and gastric cancer development

The baseline characteristics of the patients are presented in Table 2. All patients were classified using the ABC method according to the PG levels and Hp-Ab titers. Diagnostic performance for true A by serological markers alone was 100% sensitivity, 92.9% specificity, 75.8% positive predictive value, and 100% negative predictive value (Additional file 1: Table S1). The number of patients was 273 in the true A group, 87 in the pseudo A group, 518 in the B group, 515 in the C group, and 114 in the D group. The distribution of endoscopic atrophy (non /closed type /open type) in the true A, pseudo A, B, C, and D groups was 273/0/0, 0/26/61, 44/163/311, 2/28/485, and 3/4/107, respectively. In the 201 patients with Hp-Ab negative, 168 (83.6%) had open type atrophy (Table 2). In each true A, pseudo A, B, C, and D group, 0, 2, 7, 12, and 3 patients developed gastric cancer, respectively.

**Table 2:** Baseline characteristics of the patients stratified by ABC classification

Group	True A	Pseudo A	B	C	D
Total patients	273	87	518	515	114
Endoscopic atrophy non/closed/open	273/0/0	0/26/61	44/163/311	2/28/485	3/4/107
Person-years	1453.25	467.5	2560.75	2531.75	593.25
Age, mean (SD)	53.6 (15.9)	69.7 (11.1)	62.2 (12.6)	64.9 (10.7)	69.2 (11.3)
Follow-up months, mean (SD)	64.8 (57.6)	65.4 (72.3)	60.2 (60.9)	59.4 (54.0)	63.2 (62.4)
Gastric cancer	0	2	7	12	3
Age, mean (SD)	0	71.5 (1.5)	63 (10.13)	66.1 (8.8)	72.7 (4.78)
Person-years	0	2.75	25.25	68.75	14.5
Follow-up months, mean (SD)	0	16.6 (16.1)	47.6 (33.4)	63.9 (73.0)	57.2 (29.1)
Cases/incidence rate	0	0.00043	0.0027	0.005	0.0039

*SD* standard deviation

**Fig. 1:**
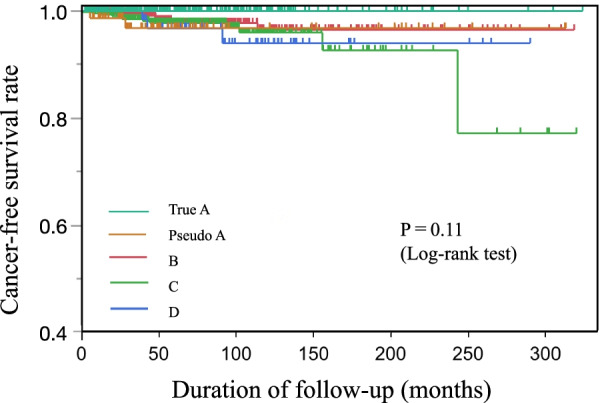
Gastric cancer-free survival rate in each group according to ABC method. Patients in group A were subclassified into the true A (without endoscopic atrophic gastritis) and pseudo A (with endoscopic atrophic gastritis) groups. Patients were classified into five groups (true A, pseudo A, B, C, and D). The differences in cancer-free survival rates among the five groups were evaluated by Kaplan–Meier analysis (log-rank test). The differences between the true A group and the other four groups were not significant (*P* = 0.11)

A Kaplan–Meier curve was generated to show differences in cancer development among the groups (Fig. 1). After a follow-up period of up to 323.7 months, no patients in the true A group developed gastric cancer; patients in all other groups, including the pseudo A group, developed gastric cancer. However, there was no statistically significant difference in carcinogenesis among those groups (log-rank test, *P* = 0.11). The cancer incidence rates for the true A, pseudo A, B, C, and D groups were 0/100,000 person-years, 43/100,000 person-years, 270/100,000 person-years, 500/100,000 person-years, and 390/100,000 person-years, respectively (Table 2). However, there were no significant differences among the five groups.

### Comparison of gastric cancer development between group A and other groups

**Fig. 2:**
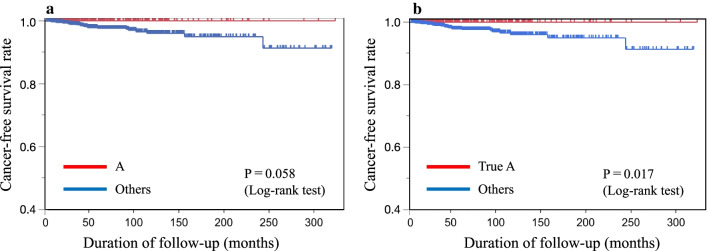
Comparison of gastric cancer-free survival rate in each group according to ABC method. **a** Patients were classified into two groups (A and others). The differences in cancer-free survival rates among the two groups were evaluated by Kaplan–Meier analysis (log-rank test). The differences between group A and others were not significant (*P* = 0.058).** b** Gastric cancer-free survival rate for the true A group and the other groups assessed using Kaplan–Meier analysis. Patients in group A were subclassified into the true A (without endoscopic atrophic gastritis) and pseudo A (with endoscopic atrophic gastritis) groups. The patients were classified into two groups (true A and others). The differences in cancer-free survival rates among the two groups were evaluated by Kaplan–Meier analysis (log-rank test). The differences between the true A group and the others group were significant (*P* = 0.017)

We categorized patients into two groups, group A and the “others” group, and performed a Kaplan–Meier analysis (Fig. 2a). There were 360 patients in group A and 1147 in the others group. The mean (standard deviation [SD]) age of the participants at the beginning of the study was 57.5 (16.4) years for group A and 64.1 (11.8) years for the others group, and the mean (SD) follow-up periods were 64.9 (61.3) and 60.1 (58.0) months, respectively (Table 3). The Kaplan–Meier analysis of the participants in the two groups showed that the incidence of gastric cancer was lower in group A than in the others group; however, the difference did not reach statistical significance (*P* = 0.058) (Fig. 2a).

**Table 3:** Baseline characteristics of the patients stratified into the A and other groups

Group	A	Others
Total patients	360	1147
Person-years	1920.75	5685.75
Age, mean (SD)	57.5 (16.4)	64.1 (11.8)
Follow-up months, mean (SD)	64.9 (61.3)	60.1 (58.0)
Gastric cancer	2	22
Age, mean (SD)	71.5 (2.1)	66 (9.5)
Person-years	2.75	108.5
Follow-up months, mean (SD)	16.6 (16.1)	60.9 (55.5)
Cases/incidence rate	0.001	0.0039

*SD* standard deviation

Approximately 24% of the patients in group A were subclassified into the pseudo A group. There was no cancer development in the true A group, whereas there were two cases of cancer development in the pseudo A group.

We then classified patients into two groups, the true A group and the “others” group, and assessed them using a Kaplan–Meier analysis (Fig. 2b). There were 273 patients in the true A group and 1,234 in the others group. The mean (SD) ages of participants at the start of the study in the true A group and the others group were 53.6 (15.9) years and 64.5 (11.9) years, respectively, and the mean (SD) follow-up periods were 64.8 (57.6) and 60.5 (59.1) months, respectively (Table 4). The Kaplan–Meier analysis of the participants in the two groups showed a significant difference between the two groups, wherein the true A group had a lower incidence of gastric cancer than the others group (*P* = 0.017) (Fig. 2b).

**Table 4:** Baseline characteristics of the patients stratified into the true A and others groups

Group	True A	Others
Total patients	273	1234
Person-years	1453.25	6153.25
Age, mean (SD)	53.6 (15.9)	64.5 (11.9)
Follow-up months, mean (SD)	64.8 (57.6)	60.5 (59.1)
Gastric cancer	0	24
Age, mean (SD)	0	66.5 (9.3)
Person-years	0	111.25
Follow-up months, mean (SD)	0	57.2 (54.6)
Cases/incidence rate	0	0.0039

*SD* standard deviation

## Discussion/Conclusions

This retrospective cohort study of a large number of patients revealed that the development of gastric cancer in group A was not significantly different from that in the other groups. However, when the low-risk group was strictly defined as the true A group based on serological and endoscopic evaluations, the results showed that no patients developed gastric cancer in the true A group.

As mentioned earlier, the ABC method is recognized as a valuable method for the risk stratification of gastric cancer [17–20]. In clinical practice, participants in group A are regarded as *H. pylori*-uninfected and are considered to have little risk for gastric cancer development [18, 19]. However, we previously demonstrated that approximately 10% of patients with gastric cancer were subclassified into group A [21]. The Japanese Society of Gastrointestinal Cancer Screening recommends endoscopic screening, even for patients in group A [26]. In addition, the Japanese Society of Helicobacter Research has established a proven strategy for the risk stratification of gastric cancer, whereby an Hp-Ab titer of < 3 U/mL (Eiken E-plate) and no endoscopic atrophy on screening indicate little or no risk of gastric cancer [23], and our results support this current strategy.

Though 87 patients in the pseudo A group and 114 in the D group (201 cases) were HP-Ab negative, 168/201 cases (83.6%) had open type atrophy. As many as 168 cases of open type gastritis were present in Hp-Ab negative patients who had no history of eradication. There were some cases with severe atrophy on endoscopy, even in Hp-Ab negative patients.

Recently, it was reported that approximately 10% of the patients with atrophic gastritis were regarded as having accidental/unintended eradication by antibiotics, except for intended eradication therapy [27]. This explains why some group A patients developed gastric cancer. In our study, 24% of the patients in group A were subclassified into the pseudo A group. There were no cases of gastric cancer in the true A group. However, 2 of the 87 patients in the pseudo A group developed gastric cancer, and the cases/incidence rate was 0.00043.

This study had some limitations. First, it was a retrospective cohort analysis performed in a single hospital. Second, the diagnosis of endoscopic atrophy could have differed among the different endoscopists who performed these procedures. To avoid these issues, a prospective cohort study should be conducted in multiple facilities, with the endoscopy performed by individuals with a certain level of endoscopic skills. Third, a strict evaluation of gastritis based on histological findings has not been performed. The histological evaluation could enable a more precise stratification of gastric cancer risk.

The development of gastric cancer in patients without *H. pylori* infection and endoscopic atrophy is very rare in Japan [28]. Our study also showed that the true A group, that is, the population negative for Hp-Abs and no endoscopic atrophy, had a very low risk of developing gastric cancer. However, its prevalence has recently increased along with the widespread use of representative images of gastric cancer without *H. pylori* infection, for instance, raspberry-like polyp or fundic gland-type cancer [29, 30]. Therefore, it cannot be concluded that endoscopic follow-up is unnecessary in the true A group. Future studies will need to determine the interval between endoscopies for those classified into the true A group. Patients in group A of the ABC method should also be evaluated for endoscopic atrophy, and those in the pseudo A group may need to undergo annual checkups as in the other group.

In conclusion, our results demonstrated that the combination of serum markers and endoscopic findings is essential for risk stratification of gastric cancer. This combination helps to identify people with a low risk of gastric cancer. Since a certain number of gastric cancer cases were observed even in serum marker-negative cases, it is also important to perform an endoscopic evaluation.

## Supplementary Information

**Additional file 1.** **Table S1.** Comparison of the diagnostic performance of serological markers (PG and Hp-Ab) alone and the combination of serological markers and endoscopic atrophy for tureA.

## Data Availability

All data generated or analyzed during this study are included in this published article.

## Abbreviations

PGs: Pepsinogens
Hp-Ab: Anti-*Helicobacter pylori* antibody
SD: Standard deviation
