# Supplementary material for: Identification of Helicobacter pylori-related gastric cancer risk using serological gastritis markers and endoscopic findings: a large-scale retrospective cohort study
Source: BMC Gastroenterol. 2022 Jun 20;22:299. doi: 10.1186/s12876-022-02381-z (PMC9210732; doi:10.1186/s12876-022-02381-z)
Supplement: Supplementary file 1 — Additional file 1. Table S1. Comparison of the diagnostic performance of serological markers (PG and Hp-Ab) alone and the combination of serological markers and endoscopic atrophy for tureA. [file 12876_2022_2381_MOESM1_ESM.pdf]

Supplementary Table 1. Comparison of the diagnostic performance of serological markers (PG and Hp-Ab) alone and the combination of serological markers and endoscopic atrophy for tureA

	Serological markers	Serological markers and endoscopic atrophy
Sensitivity (%)	100	100
Specificity (%)	92.9	100
Positive predictive value (%)	75.8	100
Negative predictive value (%)	100	100
